# Current Status of Generative Artificial Intelligence Utilization in Medical Education: A Cross-Sectional Survey of Medical Students and Faculty

**DOI:** 10.31662/jmaj.2025-0580

**Published:** 2026-04-24

**Authors:** Shigeo Ninomiya, Kyoko Yamamoto, Eiko Mieno, Hirofumi Anai, Naoto Uemura, Takashi Kobayashi, Masato Tanigawa, Takashi Hirano, Isao Saito, Toshikatsu Hanada, Yuichi Endo, Yusuke Matsunobu, Tatsushi Tokuyasu, Kenji Ihara, Masafumi Inomata

**Affiliations:** 1 Department of Gastroenterological and Pediatric Surgery, Oita University Faculty of Medicine, Yufu, Japan; 2 School of Medicine, Oita University Faculty of Medicine, Yufu, Japan; 3 School of Nursing, Oita University Faculty of Medicine, Yufu, Japan; 4 Department of Advanced Medical Sciences, Oita University Faculty of Medicine, Yufu, Japan; 5 Oita University Faculty of Medicine, Yufu, Japan; 6 Department of Otorhinolaryngology & Head and Neck Surgery, Oita University Faculty of Medicine, Yufu, Japan; 7 Department of Public Health and Epidemiology, Oita University Faculty of Medicine, Yufu, Japan; 8 Department of Biochemistry and Molecular Genetics, Oita University Faculty of Medicine, Yufu, Japan; 9 Department of Healthcare AI Data Science, Oita University Faculty of Medicine, Yufu, Japan; 10 Department of Information Systems and Engineering, Faculty of Information Engineering, Fukuoka Institute of Technology, Fukuoka, Japan; 11 Oita University Hospital, Yufu, Japan; 12 Oita University Faculty of Medicine, Yufu, Japan

**Keywords:** artificial intelligence, generative artificial intelligence, medical education

## Abstract

**Introduction:**

Generative artificial intelligence (gAI), particularly large language models such as ChatGPT, is rapidly transforming various sectors, including medical education. Despite increasing interest, few studies have investigated how gAI is actually used in medical education settings, especially in Japan. This study aimed to assess the current use of gAI among medical students and faculty members, and to compare gAI usage patterns and attitudes between these two groups.

**Methods:**

A cross-sectional survey was conducted from April to May 2025 at the Oita University Faculty of Medicine. A total of 1,017 students and 470 faculty members from the School of Medicine, School of Nursing, and Department of Advanced Medical Sciences were invited to complete an anonymous online questionnaire. The survey covered gAI usage experience, purposes of use, and attitudes toward gAI in academic contexts.

**Results:**

The response rates were 40% for students (402/1,017) and 74% for faculty members (350/470). Most students (82.1%) and faculty (73.4%) had prior experience using gAI tools, primarily for report writing, lecture preparation, and information retrieval, with students showing a higher rate of gAI usage experience than faculty (p < 0.05). While 92.8% of students and 86.5% of faculty supported gAI use under certain conditions, 73.4% of faculty members reported major concerns, including ethical risks and the risk of personal information leakage. In the faculty survey, younger faculty members, and those with gAI usage experience were also significantly more likely to approve the introduction of gAI into medical education (p < 0.05).

**Conclusions:**

gAI is widely accepted and used in medical education. However, ethical guidelines, digital literacy education, and thoughtful integration strategies are essential to ensure its responsible use.

## Introduction

Generative artificial intelligence (gAI) is defined as a class of AI models that generate synthetic outputs based on learning acquired from the datasets used to train the model ^(1)^. Among these, ChatGPT, one of the most well-known models, has gained explosive popularity, reaching 100 million users within just 2 months of its release in 2022 ^(2)^. In recent years, gAI has brought transformative changes to a wide range of fields, including scientific research, creative arts, customer service, personalized learning, and healthcare. However, research on the actual use of gAI in medical education in Japan has been limited.

Recently, medical students are increasingly believed to be using gAI for various academic tasks, such as attending lectures and writing reports. Conversely, faculty members are thought to use gAI for preparing teaching materials and drafting documents. Despite its benefits, the use of gAI in medical education presents several challenges. First, gAI systems are not always accurate and may provide outdated or incorrect information, which can mislead students and hinder their learning ^(3)^. Additionally, excessive reliance on gAI tools may impair the development of critical thinking and problem-solving skills by encouraging dependency on automated answers ^(3)^. Ethical concerns also arise, particularly regarding privacy and data security, as gAI systems often require access to sensitive personal information ^(4)^. Moreover, gAI algorithms can reflect biases in their training data, potentially reinforcing unfairness, or misinformation in educational content. Finally, overuse of gAI may reduce meaningful human interaction between students and instructors, which is vital for nurturing communication skills and professional judgment ^(5)^.

Given this social context, the present study aimed to investigate the current use of gAI among medical students and faculty members, to identify potential concerns or challenges, and to compare gAI usage patterns and attitudes between these two groups to clarify similarities and differences that may inform the future integration of gAI into medical education.

## Materials and Methods

Between April and May 2025, a cross-sectional survey was conducted among medical students and faculty members at the Oita University Faculty of Medicine. All eligible students and faculty members across all academic years and departments were invited to participate via an institutional email mailing list. Participation was entirely voluntary, no specific inclusion or exclusion criteria were applied, responses were collected anonymously, and no incentives were provided for completing the questionnaire. The questionnaire comprised nine items each for students and faculty members and was created using Google Forms and administered online. It was developed based on a literature review and under the supervision of experts in medical education and AI (TT and YM), and the full questionnaire is provided as a Supplementary File. The questionnaire was intentionally designed to be exploratory and descriptive in nature to capture an overall picture of gAI use and related attitudes. As survey items were analyzed individually rather than as composite scales, formal psychometric validation, including reliability testing, was not conducted, as it was not considered applicable. To ensure content validity, all items were reviewed for clarity and relevance through internal discussion among the authors and by domain experts.

### Student survey: Experience and awareness of gAI use among medical students

#### Participants

The survey targeted 1,017 students enrolled in three departments at our university: the School of Medicine, the School of Nursing, and the Department of Advanced Medical Sciences.

#### Objectives

The student questionnaire included items on prior experience using gAI, specific applications used, purposes for which gAI was employed, perceptions of gAI use in university classes and assignments, experience attending classes incorporating gAI, and opinions on the appropriateness of such gAI-integrated classes.

### Faculty survey: gAI use, attitudes, and concerns regarding integration into medical education

#### Participants

The survey targeted 470 faculty members from the School of Medicine, the School of Nursing, and the Department of Advanced Medical Sciences.

#### Objectives

This survey aimed to assess whether faculty members use gAI in the preparation of lectures and educational materials and to explore their opinions on the appropriateness of student use of gAI for academic activities such as coursework and report writing. In addition, we also investigated concerns regarding the future implementation of gAI in medical education.

### Statistical analysis

We examined whether there was a significant difference in gAI usage experience between medical students and faculty members. In the faculty survey on the implementation of gAI into medical education, statistical analyses were also conducted to evaluate differences by gAI usage experience and age. Statistical analyses were performed using SPSS (version 29). The chi-square test was applied, with the significance level set at p < 0.05. Multiple comparisons were performed using the chi-square test with Bonferroni correction. Multivariable analyses were not performed because this study was exploratory in nature and the sample size was insufficient to support stable multivariable modeling.

## Results

### Student survey: Experience and awareness of gAI use among medical students

#### Response rate

The demographic characteristics of the respondents are summarized in Table 1. Only the School of Medicine includes fifth- and sixth-year levels. By gender, 236 respondents were female (58.7%), 106 were male (26.4%), and 60 did not disclose their gender (14.9%). The overall response rate among students in the Faculty of Medicine was 40% (402/1,017), with response rates of 29% (192/652) in the School of Medicine, 65% (167/257) in the School of Nursing, and 37% (41/108) in the Department of Advanced Medical Sciences. It is worth noting that the Department of Advanced Medical Sciences was established only 3 years ago, with an annual enrollment of 35 students, resulting in a smaller total student population.

**Table 1.** Response Rates and Demographic Characteristics of Medical Student Respondents by Department.

Department	Eligible students, n	Respondents n (%)	Year n (%)	Gender (M/F)
Medicine	652	192 (29)	Year 1:166 (41.3)	87/67
			Year 2: 49 (12.2)	
			Year 3: 82 (20.4)	
			Year 4: 63 (15.7)	
			Year 5: 23 (5.7)	
			Year 6: 19 (4.7)	
Nursing	257	167 (65)	Year 1: 59 (35.3)	12/138
			Year 2: 20 (11.9)	
			Year 3: 58 (34.7)	
			Year 4: 30 (17.9)	
Advanced medical sciences	108	43 (37)	Year 1: 31 (72.1)	7/31
			Year 2: 5 (11.6)	
			Year 3: 7 (16.2)	
Total	1,017	402 (40)	Not applicable	106/236

Non-respondents: School of Medicine, n = 38; School of Nursing, n = 17; Department of Advanced Medical Sciences, n = 5. F: female; M: male.

#### gAI usage experience

**Figure 1.**
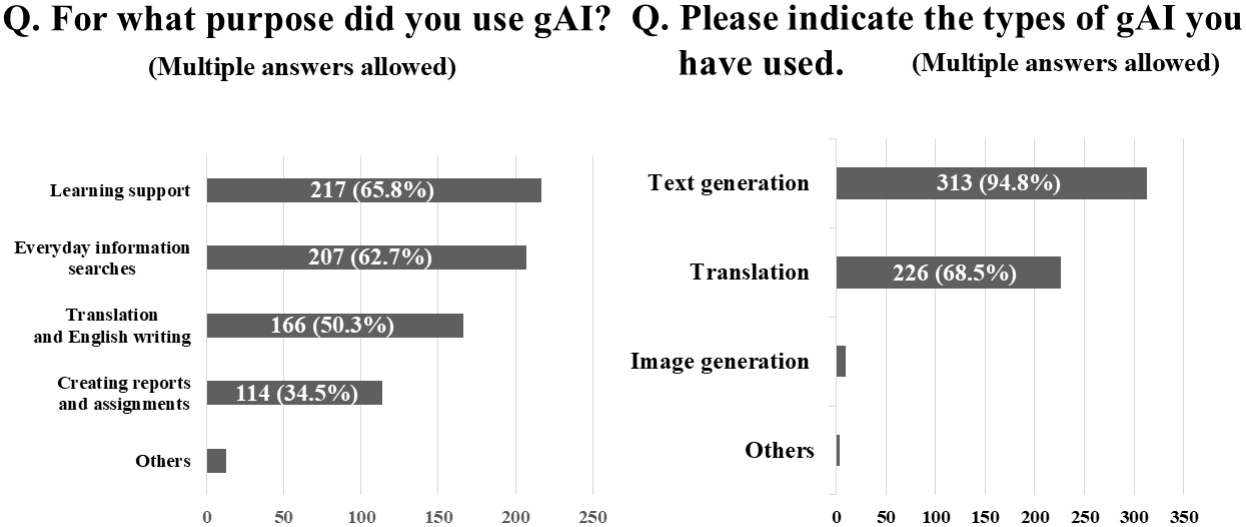
Purposes of gAI use and types of gAI used by medical students. gAI: generative artificial intelligence.

A total of 330 students (82.1%) reported having prior experience using gAI. The most frequently reported purpose of gAI use among medical students was learning support, and the most commonly used type of gAI, as indicated in the survey, was text-generating AI (Figure 1).

#### Attitudes toward gAI

**Figure 2.**
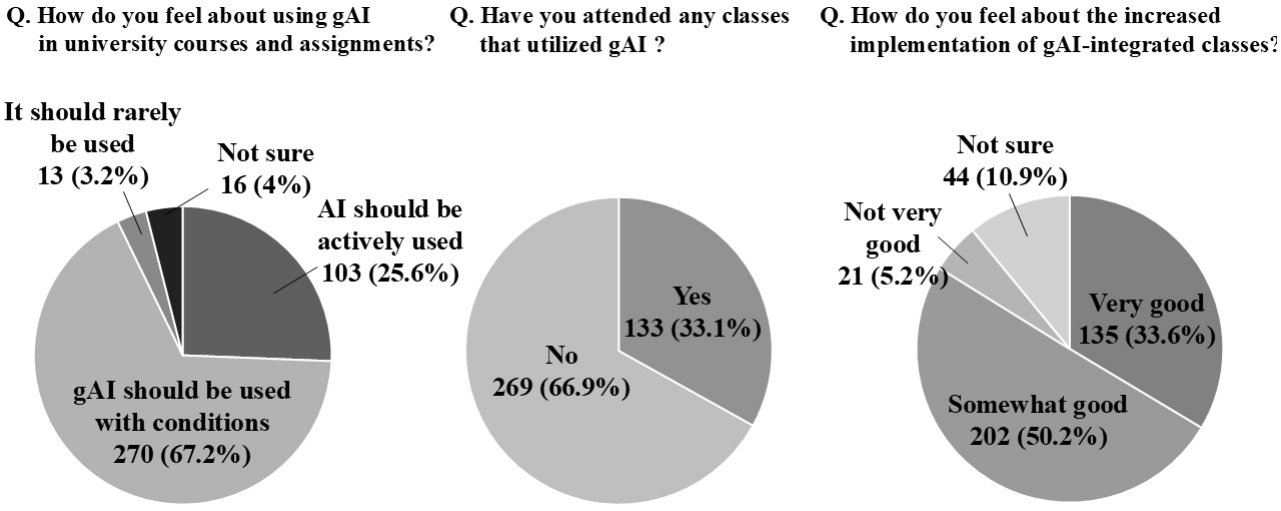
Medical students’ attitudes toward the use of gAI in university courses and assignments. gAI: generative artificial intelligence.

The attitude of medical students toward gAI use is shown in Figure 2. In response to the question, “How do you feel about using gAI in university courses and assignments?”, 103 students (25.6%) answered that gAI should be actively used, and 270 students (67.2%) responded that it should be used under certain conditions. When asked, “Have you attended any classes that used gAI?,” 133 students (33.1%) answered “Yes”. Regarding increased implementation of gAI-integrated classes, 135 students (33.6%) said it was a very good trend, while 202 students (50.2%) considered it somewhat good.

### Faculty survey: gAI use, attitudes, and concerns regarding integration into medical education

#### Response rate

The demographic characteristics of the faculty respondents are summarized in Table 2.

**Table 2.** Demographic Characteristics of Faculty Respondents.

Age group (years)	n (%)
20-29	53 (15.1)
30-39	108 (30.9)
40-49	94 (26.9)
50-59	70 (20)
≥60	25 (7.1)
Gender	n (%)
Male	176 (50.2)
Female	83 (23.8)
Not-disclosed	91 (26)
Academic position	n (%)
Professor	49 (14)
Associate professor	30 (8.6)
Lecturer	38 (10.9)
Assistant professor	92 (26.3)
Other	141 (40.2)

The response rate among faculty members was 74% (350/470).

#### gAI usage experience

**Figure 3.**
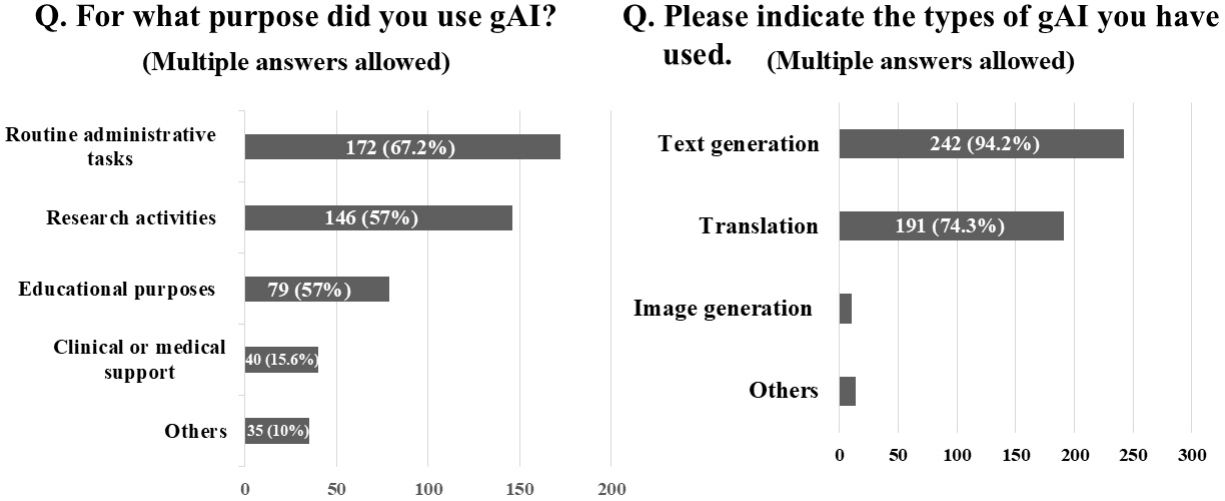
Purposes of gAI use, and types used by faculty members. gAI: generative artificial intelligence.

A total of 257 faculty members (73.4%) reported previous use of gAI, which was significantly lower than the rate of 82.1% reported by students (p < 0.05). The primary purpose of use was routine administrative tasks, followed by research and educational activities. Consistent with the findings among medical students, text-generating AI tools were the most frequently used, with a usage rate of 94.2% (Figure 3).

#### Attitudes toward gAI

**Figure 4.**
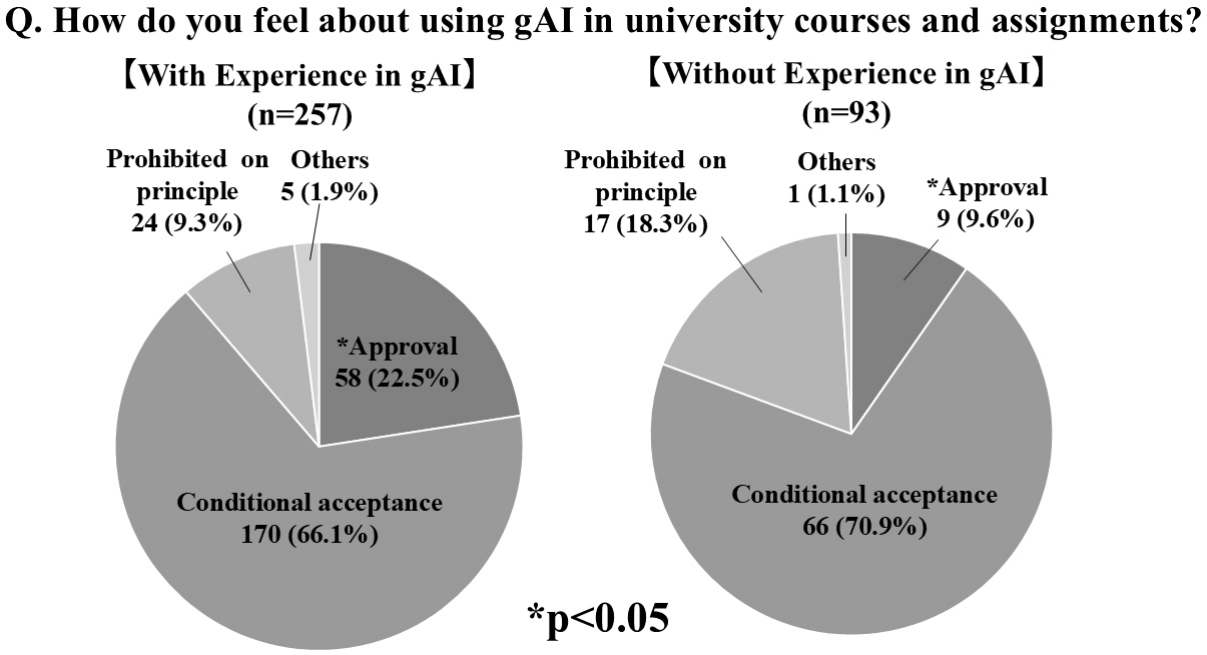
Comparison of faculty members’ attitudes toward the use of gAI in university courses and assignments, according to prior gAI usage experience. gAI: generative artificial intelligence.

**Figure 5.**
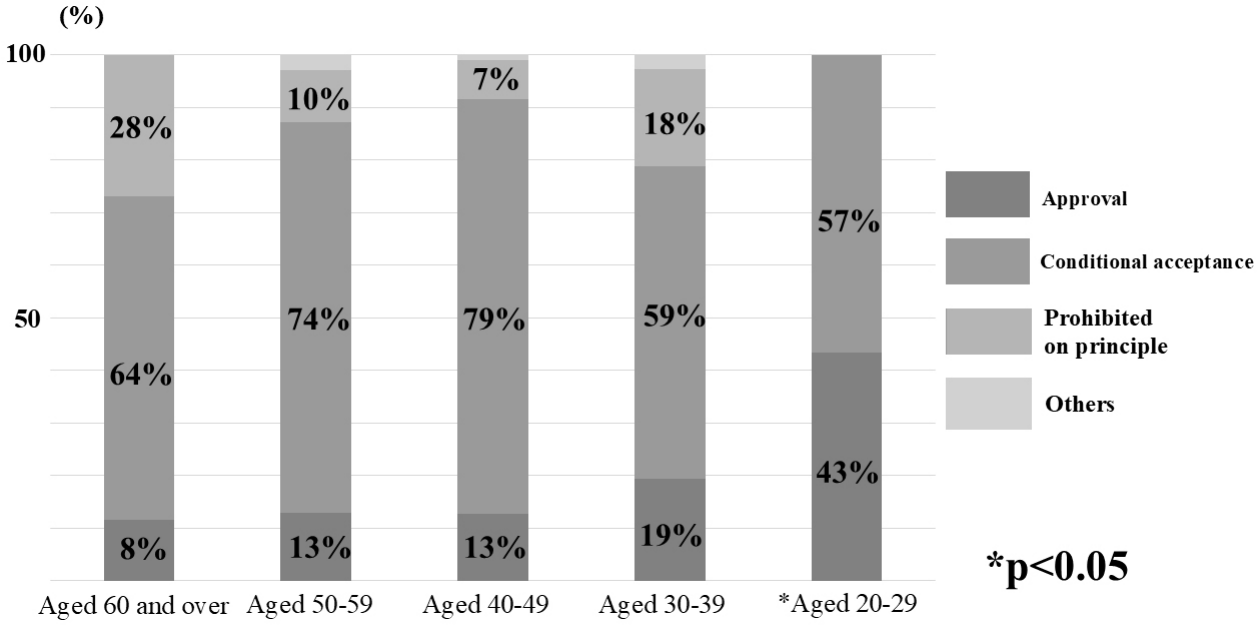
Comparison of faculty members’ attitudes toward the use of generative artificial intelligence in university courses and assignments, by age group.

**Figure 6.**
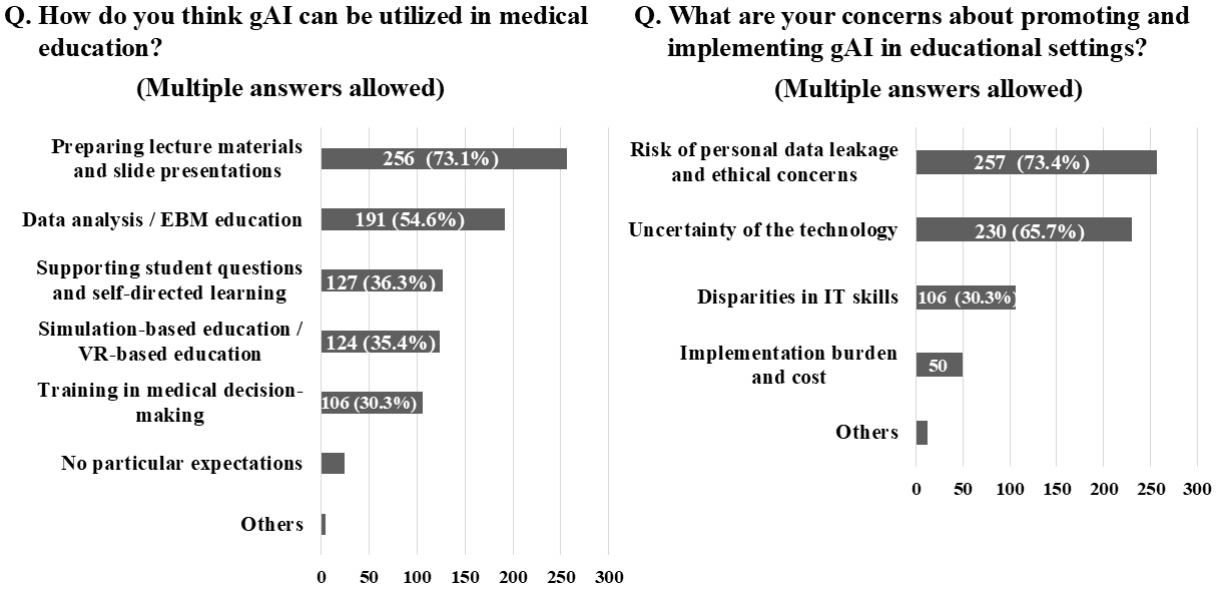
Potential applications of gAI in medical education and associated concerns raised by faculty members. gAI: generative artificial intelligence.

In response to the question, “How do you feel about using gAI in university courses and assignments?” 67 faculty members (19.1%) indicated their approval of its use, and 236 faculty members (67.4%) indicated their conditional acceptance of its use. However, 41 respondents (11.7%) believed that its use should be prohibited in principle. When comparing responses to the question “How do you feel about using gAI in university courses and assignments?” based on faculty members’ gAI usage experience, those with gAI usage experience were significantly more likely to approve the use of gAI (p < 0.05) (Figure 4). In addition, when analyzed by age group, faculty members in their 20s were significantly more likely to approve the implementation of gAI into medical education (p < 0.05) (Figure 5). Regarding how gAI could be used in medical education, the most common response was to improve the efficiency of lecture material and slide preparation (256 respondents, 73.1%), followed by its use in data analysis and evidence-based medicine education (191 respondents, 54.6%). Regarding concerns about gAI in medical education, 257 respondents (73.4%) cited issues such as the risk of personal data leakage and ethical concerns (Figure 6).

## Discussion

This cross-sectional study investigated the current status of gAI utilization and awareness among medical students and faculty members at a single Japanese university. Sami et al. ^(6)^ conducted a survey of 702 medical students in Pakistan and found that the majority perceived gAI as an effective and credible learning tool, highlighting its potential to optimize study time, improve conceptual understanding, and provide accurate medical information. Also, Abdelhafiz et al. ^(7)^ reported that Egyptian medical students showed strong interest and trust in using ChatGPT and similar chatbots for academic purposes. Subsequently, reports from various countries have described the use of gAI in medical education, with many demonstrating generally favorable attitudes toward the integration of gAI into medical education ^(6), (7), (8), (9), (10), (11), (12), (13), (14)^. To the best of our knowledge, our study is the first report from Japan. Moreover, there have been no reports that clarify the use of gAI or attitudes toward it among not only medical students but also faculty members at medical schools. Consistent with prior findings, Japanese medical students have already been using gAI in their studies and expressed a desire to continue using it in the future. In contrast, a study involving 4,313 medical students from 48 countries also reported similarly positive attitudes toward the use of gAI in healthcare and medicine, and found no significant regional differences among the countries ^(15)^.

The findings revealed that a high proportion of both students (82.1%) and faculty members (73.4%) had prior experience using gAI. These results suggest that gAI technologies have already become familiar tools in academic settings and that their integration into medical education is progressing rapidly. In contrast, the faculty group showed a slightly lower usage rate than students, possibly reflecting generational differences in digital literacy or concerns over accuracy, ethics, and educational validity. Moreover, younger faculty members with experience using gAI tended to approve of the introduction of gAI into medical education. It is anticipated that as the number of young faculty members with gAI experience increases, the implementation of gAI in medical education will be further promoted.

As mentioned previously, there were many positive opinions regarding the use of gAI in medical education. However, there are also several risks associated with integrating gAI into medical education. The first issue is ethical considerations and the risk of leakage of personal information ^(3)^. Medical education, in particular, frequently involves handling real patient information, making it essential to carefully consider the risk of personal data leakage through the careless use of gAI. In our survey of faculty members, concerns about personal information leakage and ethical risks were the most frequently cited, with 257 respondents (73.4%) expressing such concerns. In their scoping review, Gordon et al. ^(4)^ also emphasized the need to establish ethical guidelines in this area. Moving forward, it is crucial to develop clear ethical standards for the use of gAI in medical education. In contrast, concerns have been raised about skill deterioration due to overreliance on gAI and the widening digital divide among educational institutions ^(3)^. It is essential to explore effective ways to use gAI to ensure the delivery of high-quality medical education.

This study has several limitations. First, it was conducted at a single medical institution, which may limit the generalizability of the findings to other educational settings or countries. Second, the questionnaire was intentionally designed to be simple and exploratory to facilitate broad participation and to capture an overall picture of gAI use and attitudes. Consequently, the items were analyzed individually rather than as composite scales, and formal psychometric validation, including reliability testing, was not performed. This may limit the depth with which complex perceptions or attitudes toward gAI could be assessed. Third, participation in the survey was voluntary, raising the possibility of self-selection bias. Individuals with greater interest in or more favorable attitudes toward gAI may have been more likely to respond, potentially leading to an overestimation of gAI usage and positive perceptions. In addition, the response rate among medical students, particularly those in the School of Medicine, was relatively low, and non-response bias cannot be excluded. Fourth, although students from three departments―the School of Medicine, the School of Nursing, and the Department of Advanced Medical Sciences―were enrolled in the study, student data were analyzed in an aggregated manner. As curricula, educational environments, and exposure to digital technologies may differ across departments, the lack of stratified analyses among student groups may have obscured department-specific differences in gAI usage patterns and attitudes. In addition, the distribution of student respondents was skewed toward first-year students, with underrepresentation of senior students, which may further limit the generalizability of the findings, as patterns of gAI use and educational needs may vary substantially by academic year. Finally, statistical analyses were primarily limited to univariable comparisons. Multivariable analyses were not performed because of the exploratory nature of the study and considerations related to sample size and potential overfitting. Therefore, the findings should be interpreted as descriptive and hypothesis-generating. Future studies with larger and more diverse samples should employ stratified and multivariable analyses to better control for potential confounders and to more precisely evaluate factors associated with gAI use and attitudes in medical education.

In conclusion, the use of gAI in medical education is advancing, and both students and faculty members generally support its implementation under certain conditions. However, unresolved issues, particularly in the realm of ethics, must be addressed to ensure its responsible and effective implementation.

## Article Information

### Acknowledgments

We sincerely thank Rise Japan, LLC for expertly editing the English language of this manuscript.

### Author Contributions

Study concept: Shigeo Ninomiya and Masafumi Inomata. Study design: Shigeo Ninomiya and Masafumi Inomata. Data collection: Kyoko Yamamoto, Eiko Mieno, Hirofumi Anai, Naoto Uemura, Takashi Kobayashi, Masato Tanigawa, and Takashi Hirano. Data analysis: Isao Saito, Toshikatsu Hanada, Yuichi Endo and Kenji Ihara. Supervision: Yusuke Matsunobu and Tatsushi Tokuyasu.

### Conflicts of Interest

Masafumi Inomata has financial conflicts of interest (Olympus Co. Ltd., SB KAWASUMI Co. Ltd., and Aderance Co. Ltd.). In addition, he serves on the editor of this journal. The other authors declare no conflicts of interest in relation to this article.

### Ethics Approval and Consent to Participate

This study was approved by the Ethics Committee of Oita University Faculty of Medicine (Approval No. 3214) and conducted in accordance with the Declaration of Helsinki. Written informed consent was waived by the committee in accordance with Japanese national regulations, as participation was based on an opt-out method. Information about the study was disclosed on the institution’s website and bulletin boards, and only data from individuals who did not opt out were included in the analysis.

### Disclaimer

Masafumi Inomata is a member of the Editorial Board of this journal but was not involved in the editorial decision or peer review of this manuscript.

## Supplement

Supplementary Material
