# Supplementary material for: Current Status of Generative Artificial Intelligence Utilization in Medical Education: A Cross-Sectional Survey of Medical Students and Faculty
Source: JMA J. 2026 Apr 24;9(4):742–9. doi: 10.31662/jmaj.2025-0580 (PMC13453582; doi:10.31662/jmaj.2025-0580)
Supplement: Supplementary file 1 [file 2433-3298-9-4-0742-s001.pdf]

Supplementary File 1. Student survey (English version)

Q1. What is your academic year?
1. 1st year 2. 2nd year 3. 3rd year 4. 4th year 5. 5th year 6. 6th year

Q2. What is your gender ?
1. Male 2. Female 3. Prefer not to answer

Q3. Which department are you affiliated with?
1. School of Medicine
2. School of Nursing
3. Department of Advanced Medical Science

Q4. Have you ever used AI tools such as ChatGPT or Copilot?
1. Yes 2. No

Q5. For what purpose did you use AI? (Multiple answers allowed)
1. Learning support
2. Everyday information searches
3. Translation and English writing
4. Creating reports and assignments
5. Others

Q6. Please indicate the types of AI you have used. (Multiple answers allowed)
1. Text generation (e.g., ChatGPT/Claude/Copilot/Gemini）
2. Translation (e.g., DeepL/Google Translate）
3. Image generation (e.g., Midjourney/Stable Difuusion）
4. Others (please specify)

Q7. How do you feel about using AI in university courses and assignment?
1. AI should be actively used.
2. AI should be used with conditions.
3. It should rarely be used.
4. Not sure

Q8. Have you attended any classes that utilized AI?
1. Yes 2. No

Q9. How do you feel about the increased implementation of AI-integrated classes?
1. Very good
2. Somewhat good
3. Not very good
4. Not sure

Supplementary File 2. Faculty survey (English version)

Q1. What is your age group?

1. 20s 2. 30s 3. 40s 4. 50s 5. 60s or older 6. Prefer not to answer

Q2. What is your gender?

1. Male 2. Female 3. Prefer not to answer

Q3. What is your current academic positions?

1. Professor 2. Associate Professor 3. Assistant Professor 4. Lecturer 5. Others

Q4. Have you ever used AI tools such as ChatGPT or Copilot?

1. Yes 2. No

Q5. For what purpose did you use AI? (Multiple answers allowed)

1. Routine administrative tasks
2. Research activities
3. Educational purposes
4. Clinical or medical support
5. Others

Q6. Please indicate the types of AI you have used. (Multiple answers allowed)

1. Text generation (e.g., ChatGPT/Claude/Copilot/Gemini）
2. Translation (e.g., Deep L/Google Translate）
3. Image generation (e.g., Midjourney/Stable Diffusion）
4. Others (please specify)

Q7. How do you feel about using AI in university courses and assignments?

1. Approval
2. Conditional acceptance
3. Prohibited on principle
4. Others

Q8. How do you think AI can be utilized in medical education? (Multiple answers allowed)

1. Preparing lecture materials and slide presentations
2. Data analysis/EBM education
3. Supporting student questions and self-directed learning
4. Simulation-based education/VR-based education
5. Training in medical decision-making
6. No particular expectations
7. Others

Q9. What are your concerns about promoting and implementing AI in medical settings? (Multiple answers allowed)

1. Risk of personal data leakage and ethical concerns
2. Uncertainty of the technology
3. Disparities in IT skills
4. Implementation burden and cost
5. Others
